# Prevalence and direct costs of vestibular disease in France – matched case-control study results from the 2022 and 2023 french health data system

**DOI:** 10.1186/s12962-026-00773-6

**Published:** 2026-05-20

**Authors:** Eva Grill, Vincent Darrouzet, Xavier Dubernard, Dimitri Parise, Stéphane Sanchez, Andreas Zwergal, Christian Chabbert

**Affiliations:** 1 Institute for Medical lnformation Processing, Biometry and Epidemiology, LMU Medizin, Ludwig-Maximilians-Universität München, Munich, Germany; 2 German Center for Vertigo and Balance Disorders, LMU University Hospital, LMU Medizin, Ludwig-Maximilians Universität München, Munich, Germany; 3 Research Centre in Psychology and Neuroscience UMR7077, CNRS-Aix Marseille University, Marseille, France; 4 University of Bordeaux, Bordeaux, France; 5 Institut Oto Neurochirurgical de Champagne Ardenne, Service d’Oto-rhino-laryngologie et de Chirurgie Cervico-Faciale, Laboratoire d’Anatomie de la Faculté de Médecine de Reims, Reims, France; 6 Expert Système National des Données de Santé, Agence Régionale de Santé Grand Est, Nancy, France; 7 Hôpitaux Champagne Sud, Pôle territorial Santé Publique et Performance, Unité de Recherche Clinique, Troyes, France; 8 UFR de Médecine, Université de Reims Champagne-Ardenne, Reims, France; 9 Department of Neurology, LMU University Hospital, LMU Medizin, Ludwig-Maximilians Universität München, Munich, Germany; 10 Institut de Recherche Equilibre et Vertige (IREV), Unit GDR2074 CNRS, Marseille, France

**Keywords:** Vestibular dysfunction, Healthcare costs, Prevalence of vestibular disorders, Betahistine

## Abstract

**Importance:**

Vestibular dysfunction causes vertigo, dizziness, and imbalance, impairing daily life and leading to substantial healthcare use. Precise prevalence and economic impact remain unclear.

**Objective:**

To estimate the one-year prevalence and direct healthcare costs of vestibular disorders in France.

**Design:**

A matched case-control study using the French national health data system covering over 67 million records in 2022.

**Setting:**

Nationwide health insurance data including all public and private healthcare interactions.

**Population:**

Adults aged ≥18 years diagnosed or treated for vestibular disease, matched 1:1 with controls by age, sex, comorbidities, region, and deprivation.

**Exposure:**

Vestibular disorder diagnosis or treatment in 2022.

**Outcome:**

Direct healthcare expenditures in 2022 and 2023.

**Results:**

Vestibular disorders affected 2.5% of adults (1,279,966 cases). Cases incurred mean costs of €4,233 versus €3,719 in controls in 2023, with an excess of €657 million nationally.

**Conclusions and relevance:**

Vestibular disorders are common and associated with significant excess healthcare costs. Standardizing diagnostic and therapeutic pathways may reduce expenditures and improve care quality.

**Supplementary Information:**

The online version contains supplementary material available at 10.1186/s12962-026-00773-6.

## Background

The vestibular system plays a critical role in human health, enabling spatial orientation, visual stability, and dynamic balance during movement. Acute or chronic vestibular dysfunction manifests as dizziness, vertigo, and imbalance, impairing daily functioning and contributing to mobility limitations and social isolation. In older adults, vestibular problems rank among the health conditions with the greatest impact on mobility and social participation, and they are associated with the accumulation of multiple health deficits over time [1]. Vestibular disease is a recognized risk factor for falls [2, 3] and is linked to reduced mobility, restricted community engagement [4, 5], and poorer overall health outcomes [6].

Beyond its essential role in gait and stance control, vestibular sensory input influences autonomic, i.e., neurovegetative, oculomotor, emotional, cognitive, and hormonal functions [7]. Loss of vestibular input can promote sedentary behaviour and disrupt circadian rhythm [8], sleep [9, 10], and thermoregulation [11]. Vestibular dysfunction is considered one of the most relevant modifiable risk factors for accelerated cognitive decline and dementia [12, 13]. Epidemiological studies estimate the age-adjusted prevalence of vestibular dysfunction in the general population at approximately 7% [6], with effects on metabolic health [14–16] and increased all-cause mortality risk, independent of age, sex, and comorbidities [17, 18].

The clinical burden of vestibular disorders translates into substantial healthcare use. In Germany, up to 10% of adults aged 18–74 years consult primary care annually for vertigo or dizziness [19]. However, the true prevalence and economic impact of vestibular disease remain incompletely understood. Existing cost-of-illness studies suggest a substantial economic burden: in the United States, incremental direct medical costs for three vestibular disease entities ranged from $2,087 to $5,211 per patient per year, amounting to approximately $60 billion in excess national expenditure [20]. In South Korea, 4.1% of adults sought medical care for dizziness or vertigo in 2022, generating total direct costs of $406.5 million [21]. These high costs are partly unexplained, given that common peripheral vestibular disorders are typically benign and amenable to treatment through lifestyle modification, vestibular rehabilitation, medication and physical therapy. Previous work in Germany indicates that excessive costs arise from repeated, non-targeted consultations, overuse of diagnostic imaging, and frequent emergency department visits [22], a pattern also observed in U.S. data [23].

Despite their clear relevance to public health, vestibular disorders are often perceived by decision makers as less serious than other conditions with similar prevalence, leading to lower prioritization in health policy. Consequently, vestibular disorders remain largely absent from burden-of-disease and cost-priority agendas. Therefore, robust, population-representative data are essential to fully quantify the prevalence, healthcare utilization, and costs attributable to vestibular disease. France’s National Health Data System (Système national des données de santé, SNDS) covers over 98% of the population and offers a comprehensive, validated resource for analysing healthcare utilization, diagnoses, and expenditures across all sectors [24].

### Study objectives

This study aims to (1) estimate the one-year prevalence of likely vestibular disorder cases in France, (2) compare direct healthcare costs of these patients with a matched cohort without vestibular-specific drug prescriptions, diagnostic or therapeutic procedure to quantify incremental costs, and (3) characterize patterns of healthcare utilization—including primary care, specialist visits, and hospitalizations—among patients with vestibular disease. We used the RECORD checklist for reporting.

## Methods

We conducted a matched case-control study within the entire French population aged 18 or older, from January, 1, 2022 to December, 31, 2022. Data was retrieved from the French national health data system (Système national des données de santé - SNDS) which covers > 98% of the French population. Permanent access to this data base is granted to the Centre National de Recherche Scientifique upon request. The SNDS contains data from all beneficiaries who, in a given year, had at least one contact with any public or private facility of the health system such as a primary care consultation, inpatient or outpatient hospital care, rehabilitation, or home care as part of the obligatory French health insurance (la Caisse nationale de l’assurance Maladie (CNAM)). The SNDS provides additional tables with data on expenses, which are summarised per individual beneficiary and data on presence and absence of health conditions, in a section termed ‘cartography’. EG and DP had full access to the entire data base.

### Exposed study sample (cases)

As there is no distinct information on diagnostic codes (e.g., ICD-10 codes) for outpatient care in the French SNDS, we established a selection algorithm for vestibular disease based on previous research and expert opinion. Betahistine and acetylleucine are almost exclusively prescribed for vertiginous diseases. Recent analysis of the SNDS data found that about 1% of the French population have at least one prescription of betahistine annually [25] corresponding to current estimates of the prevalence of moderate to severe vestibular disease from other countries [26]. As not all patients with vestibular disease receive medication [27], we supplemented the algorithm with diagnostic or therapeutic procedures typical for the diagnosis e.g., caloric testing, head impulse testing, posturography, or therapy of vestibular disease, e.g., vestibular rehabilitation or inner ear surgery etc. (complete list of procedure codes in supplement). The list of procedures was compiled based on expert knowledge in neuro-otology (VD, XD and AZ). This algorithm was intended to identify patients diagnosed or treated for vestibular disease in routine care rather than all persons with vestibular symptoms or vestibular dysfunction in the population. A similar algorithm was also recently applied by a previous French national health insurance analysis which also verified the link of ICD diagnoses and procedures [28].

From all patients who were beneficiaries of the French health system in metropolitan (mainland and Corsica) France in 2022 we selected individuals aged ≥ 18 years who filled in at least one prescription of betahistine (Anatomical Therapeutical Chemical - ATC - code N07CA01) or acetylleucine (ATC code N07CA04) or had at least one of the above-named diagnostic or therapeutic procedures. As some chronic neurological or psychiatric diseases may cause vertigo or postural instability, we subsequently excluded patients who had a diagnosis of chronic neurological pathologies in 2022. Excluded pathologies were Parkinson’s disease, multiple sclerosis, epilepsy, dementia, psychosis, or stroke. We also excluded all patients who died during 2022 or 2023.

### Non-exposed study sample (controls)

From all beneficiaries in 2022 we selected those adult patients who were not included in the exposed sample, using the same exclusion criteria as in the case group.

Each case was matched to one control by age, sex, comorbidity profile, region, and regional deprivation.

Comorbidity was quantified using the chronic disease and long-term condition indicators available in the SNDS cartography, which identify major treated conditions from hospital diagnoses, long-term disease status, and disease-specific drug dispensations. For matching, we used the number of long-term comorbidities derived from these indicators, together with age, sex, region, and regional deprivation. In addition, all available disease indicators were included in the regression model to account for residual differences in measured chronic disease profiles between cases and controls.

### Primary outcomes

We used the total and specific direct medical expenditures incurred during 2022 and 2023. In the SNDS cartography expenditure data used in this study, the grand total of reimbursed expenditures includes not only outpatient and inpatient healthcare costs, but also cash benefits reimbursed by the French national health insurance system, including daily sickness allowances, maternity-related benefits, occupational accident or occupational disease compensation, and disability-related benefits. This information is provided by the SNDS by expense category. It includes the data of all beneficiaries of CNAM who had at least one interaction with the health system that caused the reimbursement of expenses for CNAM (https://www.assurance-maladie.ameli.fr/etudes-et-donnees/par-theme/pathologies/cartographie-assurance-maladie). This represents 67 million beneficiaries. We used data from 2022 to 2023. The year 2023 is currently the most recent year available with complete cost data.

### Covariates

We included age in 2022 and age groups as provided by the SNDS.

The cartography provides quintiles of the National French index of regional deprivation for each patient (French Version of the European Deprivation Index, version 2020 [29]). This index, available from OpenDataSoft [30, 31], is a composite indicator of the socio-economic status at the household level, accounting for territorial disparities.

The cartography also provides indicators of the most frequent pathologies and estimates of their prevalence in a given year based on validated algorithms combining inpatient stays caused by a specific health condition, typical medication or characterization as a chronic disease. These indicators are available for diseases such as cardiovascular disease, diabetes or cancer and have been extensively validated [32].

### Statistical analyses

We used data from the French national health data system, which covers nearly the entire French population through health insurance claims and hospital records. Given the exhaustive nature of this dataset, our analyses reflect the full population rather than a sample. Statistical inference methods such as p-values or confidence intervals—used to quantify uncertainty due to sampling variability—are not applicable because in full population samples there is no sampling error. Instead, we present descriptive statistics that characterize the population directly, following established recommendations for register-based epidemiological studies using population-wide data sources [24, 33]. Thus, any differences between the cost values of cases and controls do not reflect estimates but the true difference in a given year.

To gain more insights into the determinants of costs, we formulated a log-linear regression model that contained a 1/0 indicator for cases and controls, all variables used for matching and the available indicators for other pathologies as independent variables, and total costs as dependent variable. To account for zero-inflation and skewedness of the outcome variable, we used hurdle (mixture) models with a gamma distribution for the nonzero part and a degenerate distribution centered at 0 for the zero part. Mixture models account for complex distributions that consist of more than one underlying distribution such as skewed cost data with zero inflation.

Data analysis used SAS Enterprise Guide version 8 software (SAS Institute, Cary, North Carolina). Finite mixture models were formulated using SAS proc fmm.

## Results

### Population with vestibular disease

As there is no distinct information on diagnostic codes (e.g., ICD-10 codes) for outpatient care in the French SNDS, we established a selection algorithm for vestibular disease based on previous research and expert opinion. From the entire French population aged 18 or older, from January, 1, 2022 to December, 31, 2022 we retrieved 1,595,036 patients who had either diagnostic or therapeutic procedures linked to vestibular disease or filled in a prescription for betahistine or acetylleucine in 2022. After exclusion of neurological pathologies, and patients deceased in 2022 or 2023, 1,281,958 individuals (70.1% female, mean age 61.45 years, range 18 to 110) were identified as being diagnosed or treated for vestibular disease and selected into the case group. To put this into perspective, this is 2.5% of the adult population in metropolitan France (i.e., mainland France and Corsica).

### Study population

**Fig. 1:**
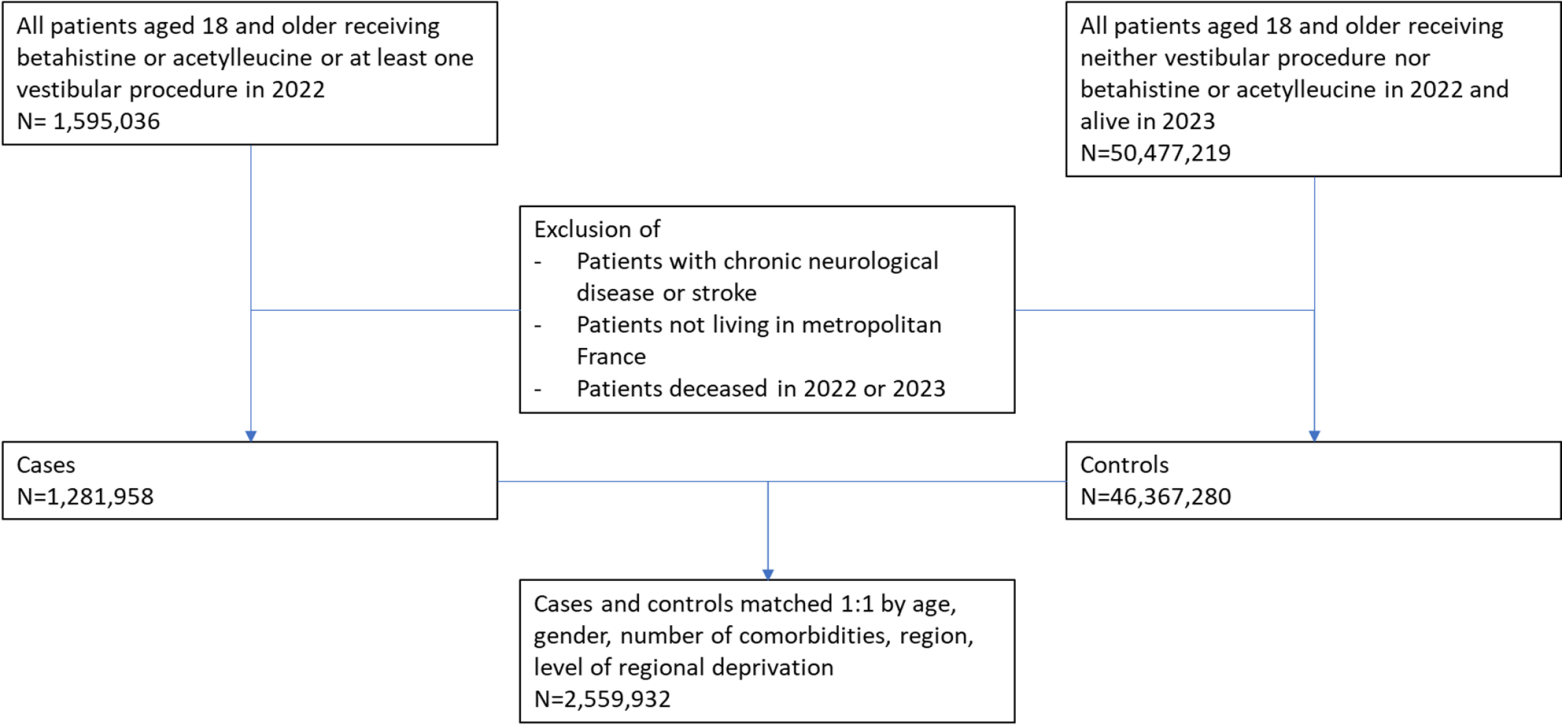
Study population

We performed exact 1:1 matching to the non-exposed sample of 46,367,280 patients using the same exclusion criteria, matching by age, gender, number of long-term comorbidities, region, and level of regional deprivation. After exclusions, 1,281,958 individuals were identified as eligible cases; of these, 1,279,966 could be matched 1:1 to a non-exposed control and therefore constituted the final analytical case population. For 1,992 exposed participants (0.15%), no matching non-exposed participants could be found (see Fig. 1 for details). All subsequent results are reported for the matched sample.

Of 1,279,966 exposed matched cases (mean age 61.44 years, standard deviation 17.82, range 18–109) 70.1% were female (Table 1). The regional distribution of cases and controls matched the population distribution of metropolitan France more or less closely, with fewer patients than expected in the Île-de-France region and more in the southern regions of Nouvelle-Aquitaine, Occitanie, and Provence-Alpes-Côte d’Azur (see Table S1 for the population distribution). Included individuals were more likely than the general population to live in deprived areas. Cases had a slightly lower prevalence of diabetes, end-stage kidney disease, pregnancy and cancer, and a slightly higher prevalence of chronic respiratory, liver, pancreatic, cardiovascular and inflammatory diseases. A total of 4,727 cases had spent at least one day in a nursing home in 2023 (controls: 8,674 persons).

**Table 1:** Sociodemographic characteristics of patients with vestibular disease (cases) and matched controls. Each case was matched to one control by age, sex, number of comorbidities (based on the list of long-term conditions that entitle patients to complete refund of medical expenditures), region and regional deprivation

	cases		controls	
	No.	%	No.	%
Variables used for 1:1 matching				
Gender Female	897,279	70.1	897,279	70.1
Age class				
18–34	115,443	9.0	115,443	9.0
35–54	315,629	24.7	315,629	24.7
55–64	234,015	18.3	234,015	18.3
65–74	277,436	21.7	277,436	21.7
75 and over	337,443	26.4	337,443	26.4
Deprivation Quintiles (2020)				
na	8,154	0.6	8,154	0.6
1 (least deprived)	219,933	17.2	219,933	17.2
2	246,992	19.3	246,992	19.3
3	261,481	20.4	261,481	20.4
4	270,578	21.1	270,578	21.1
5 (most deprived)	272,828	21.3	272,828	21.3
Region				
AUVERGNE RHONE ALPES	146,315	11.4	146,315	11.4
BOURGOGNE FRANCHE COMTE	58,162	4.5	58,162	4.5
BRETAGNE	48,793	3.8	48,793	3.8
CENTRE VAL DE LOIRE	50,970	4.0	50,970	4.0
CORSE	7,685	0.6	7,685	0.6
GRAND EST	120,161	9.4	120,162	9.4
HAUTS DE FRANCE	123,469	9.6	123,469	9.6
ILE DE FRANCE	207,880	16.2	207,880	16.2
NORMANDIE	71,350	5.6	71,350	5.6
NOUVELLE AQUITAINE	132,917	10.4	132,917	10.4
OCCITANIE	127,242	9.9	127,242	9.9
PAYS DE LA LOIRE	60,608	4.7	60,608	4.7
PROVENCE ALPES COTE D AZUR	124,414	9.7	124,415	9.7
Other disease indicators (not used for matching)				
Diabetes	149,738	11.7	147,540	11.5
Cancers	119,988	9.4	120,201	9.4
Chronic respiratory disease	118,268	9.2	92,250	7.2
Chronic terminal kidney disease	1,784	0.1	2,255	0.2
Inflammatory disease, HIV	45,343	3.5	44,100	3.4
Disease of liver or pancreas	14,666	1.1	13,397	1.0
Pregnancy and birth	12,632	1.0	19,015	1.5
Obesity treated in hospital	70,936	5.5	57,963	4.5
Pain and antiinflammatory medication without other pathologies	40,476	3.2	20,049	1.6
Cardiovascular disease	166,878	13.0	162,112	12.7

**Table 2:** Cost components for direct medical expenses in metropolitan France in 2023. Costs represent the total expenditures of the French national health insurance and do not include copayments or deductibles. All monetary values are reported in euros. Sums are shown without decimals; means and standard deviations are shown with two decimal places where applicable

	Cases		Controls	
	Sum	Mean (SD)	Sum	Mean (SD)
**Outpatient care**				
General practitioner	187,071,104	146.15 (151.51)	137,372,094	107.32 (124.41)
Specialist	373,906,012	292.12 (672.50)	292,286,587	228.35 (672.00)
Dentist	105,996,997	82.81 (176.88)	936,655,49	73.18 (164.88)
Midwives	68,999,17	5.39 (49.77)	69,989,52	5.47 (56.29)
Physical therapy	169,305,119	132.27 (374.00)	119,972,865	93.73 (332.43)
Nursing	292,740,064	228.71 (1,311.19)	277,672,994	216.94 (1,329.41)
Other health professionals^a^	164,405,31	12.84 (94.08)	108,717,76	8.49 (74.12)
Tests and imaging	104,528,672	81.67 (129.45)	89,741,840	70.11 (126.91)
Medication	880,366,505	687.80 (3,795.33)	828,241,576	647.08 (3,873.23)
Other health products	243,947,810	190.59 (689.57)	217,249,820	169.73 (746.44)
Transports	153,103,367	119.62 (862.41)	138,002,493	107.82 (889.78)
Other outpatient expenses	14,561,528	11.38 (85.42)	121,166,30	9.47 (76.55)
**Total outpatient services**	2,548,867,624	1,991.36 (4,856.47)	2,224,193,175	1,737.7 (4,930.01)
**Inpatient care**				
General hospital	1,754,730,635	1,370.92 (5,858.62)	1,666,375,077	1,301.89 (6,073.28)
Psychiatric hospital	26,951,907	21.06 (876.60)	22,734,909	17.76 (817.58)
Rehabilitation hospital	277,857,155	217.08 (2,066.77)	256,832,250	200.66 (2,113.19)
Nursing care at home	24,130,446	18.85 (1,151.18)	31,048,923	24.26 (959.40)
**Total hospital/inpatient services**	2,083,670,142	1,627.91 (6,864.35)	1,976,991,160	1,544.57 (7,045.73)
**Total expenses**	**5**,**418**,**429**,**252**	**4**,**233.26 (9**,**945.14)**	**4**,**761**,**111**,**898**	**3**,**720.00 (10**,**089.46)**

^a^orthoptists, speech therapists, podiatrists

**Fig. 2:**
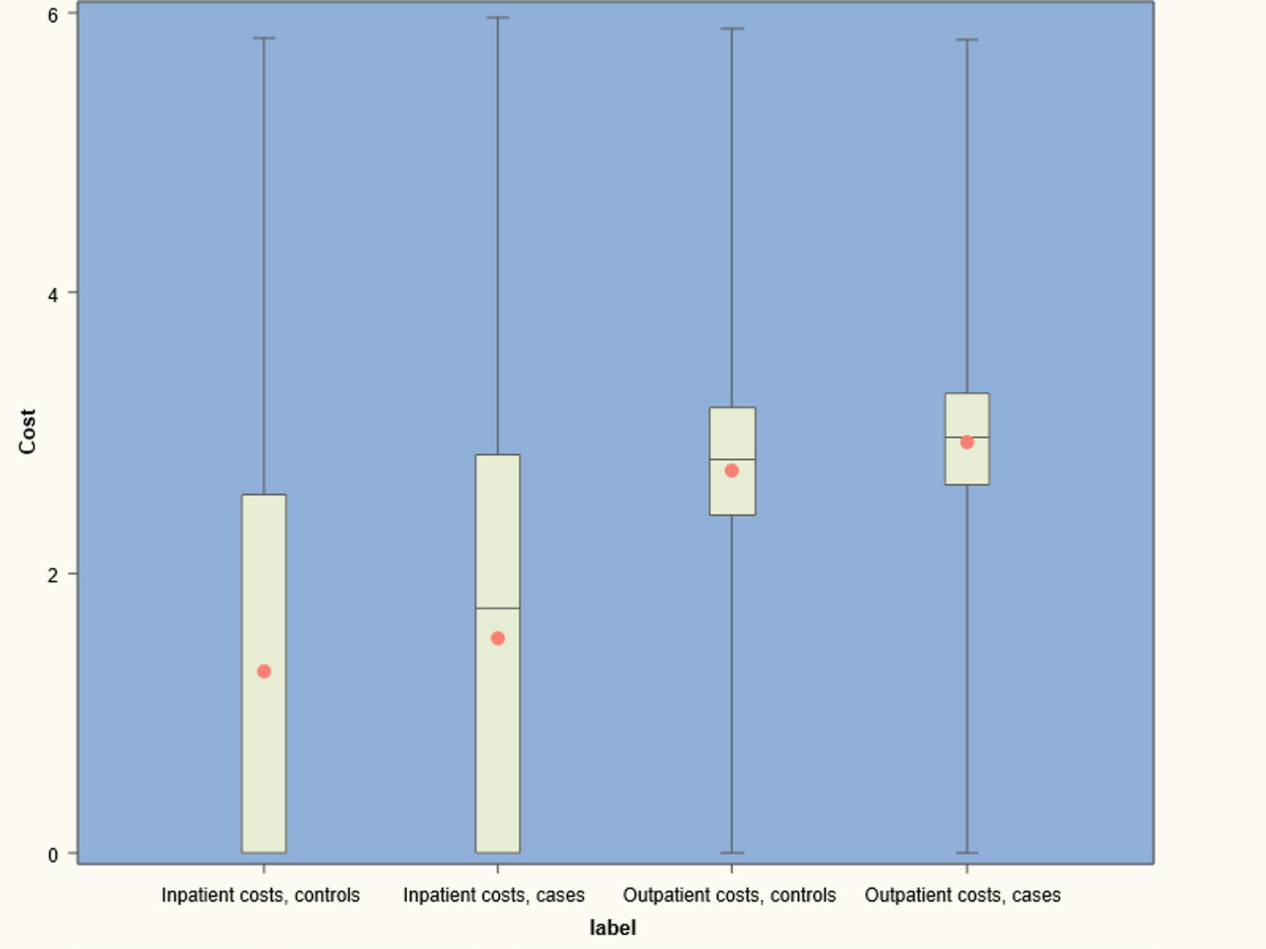
Comparison of single cost components in cases with vestibular disease and controls without vestibular disease matched 1:1 to cases by age, gender, number of comorbidities, region and level of regional deprivation; costs on the decimal logarithmic scale in Euro. The mean is indicated by bar, median by dot. The box shows the interquartile range, whiskers range from minimum to maximum value

The cost breakdown and total costs shown in Table 2 represent the total expenditure of the French national health insurance system and do not include copayments or deductibles. In 2023, a total of 12,521 cases (1.0%) had no interaction with the health system and accrued no costs (36,740 controls, 2.9%). In 2023, cases had mean costs of €4,233 per participant (controls: €3,719). Total expense of the case population was 5,42 billion Euros. Cases accrued 657.0 million Euro more in total expenditure (€514 per person) than controls in 2023. Tests and imaging accounted for incremental 14.8 million €. The main cost components were €1,991 per case for outpatient care (controls: €1,738) and €1,628 for inpatient care (controls: €1,545). Cases incurred lower costs than controls for domestic nursing care, and midwifery services. Comparisons of outpatient and inpatient costs are shown in Fig. 2.

To account for small discrepancies in the contribution of chronic comorbidities, we formulated a log-linear mixture regression model with total costs as the outcome variable, including the matching variables and all available disease indicators as independent variables. The model showed that, when all other factors were held constant, individuals with vestibular disease still incurred significantly higher annual healthcare costs than those without. For example, for a low-risk patient (a female under 35 years old in the least deprived quintile, living in the Val de Loire department without comorbidities), the model predicted significant incremental costs of €213 for a person with vestibular disease (see also supplemental table S2).

As a sensitivity analysis, we also examined expenses accrued in 2022 by cases and controls. Absolute total expenses were lower for both groups, with a mean of €3,650 for cases and €3,122 for controls. Cases accrued €675.7 million more than controls, corresponding to €528 more per person. Tests and imaging accounted for an additional €23.8 million in costs for cases compared to controls (see also supplemental table S3).

## Discussion

We found that vestibular disease is common and, in both 2022 and 2023, was associated with significantly higher medical costs compared to a matched group of patients without vestibular disease. This study is the first to comprehensively assess the true prevalence and direct medical costs of vestibular disease in the entire population of a country. The burden of these conditions on the healthcare system is considerable, with the total cost of direct care for persons with vestibular disorders amounting to around €5.5 billion, representing 2.3% of all healthcare expenditure on care and medical goods in France.

Our results show that in 2022, 1.28 million adults in France were diagnosed or treated for vestibular disease, accounting for around 2.5% of the total adult population. Estimates of the prevalence of definite vestibular disease vary widely because dedicated vestibular testing is often not feasible in large-scale studies, while studies based in clinical settings are often subject to selection and other biases. Typically, health surveys have to cope with non-response and selection bias. The German National Health Interview Survey (GNHI) found a one-year prevalence of vestibular vertigo of 4.9% and an incidence of 1.4% [34]. Their case definition was based on telephone interviews reporting symptoms, and the GNHI sample was generally younger and more highly educated than the general population. Similarly, our group found a prevalence of bilateral vestibular hypofunction of 2.5% in a sample of adults aged 39 and over from the KORA FF4 cohort study [6], based on video head impulse testing.

Analyses of routine and claims data avoid the issue of selection bias. The main strength of our study is that we could rely on validated, comprehensive, unselected data from the entire French healthcare system (SNDS). We used procedural care data and medication as indicators of vestibular disease. This approach has been validated within the SNDS for other pathologies where no International Classification of Diseases (ICD) codes are available [32]. Our approach is arguably more conservative, but it may also be more valid than using the ICD diagnoses. Firstly, it has convincingly been argued that codes of the ICD from routine data are often not suitable for research because coding is unreliable [35, 36]. Also, the ICD was found to be used in an unspecific and frequently erroneous manner in the coding of vestibular disease [19]. Secondly, primary care physicians report considerable uncertainty when faced with vestibular pathologies [37, 38], so diagnostic and therapeutical procedures and medication may be the more appropriate way to approach these pathologies. The prevalence found in our study also compares favorably with other studies. To give an example, an analysis of German claims data found that, in 2022, 1.6% of all beneficiaries of statutory German health insurance, including children and adolescents, were diagnosed with peripheral vestibular disease (ICD10 H81.-) [39]. The demographics of our study’s case population also align well with the literature. For example, we found that 70% of patients with vestibular disease were female and older, which confirms the findings of a study of primary care utilization in the United States [40] and data from the statutory German health insurance [39].

Another strength of our study is the accurate reporting of total healthcare costs, based on data from the French health insurance authorities. We found that patients with vestibular disease incurred mean incremental costs of over €500 per year, adding up to an additional total cost of €657 million. For context, French health insurance reported an average cost of €2,503 per person attributable to diabetes. In our study, cases with vestibular disease had average total expenditures of €4,233 per patient (controls: €3,719). Thus, the average expenditures for individuals with vestibular disease in our study were significantly higher than the overall mean expenditure of €2,980 per beneficiary [41]. In order to compare the costs incurred by patients with vestibular disease with those of a control population, we based our matching not only on typical sociodemographic characteristics, but also on the costliest comorbidities reflected in the long-term care status (Affection de Longue Durée, ALD) scheme. In France, this scheme ensures full coverage of healthcare costs for individuals with serious and chronic illnesses. Patients with ALD status are exempt from copayments for all care related to their specified long-term condition. Eligibility is determined by a physician and confirmed by the health insurance medical advisor based on a list of conditions defined by law (e.g., diabetes, cancer and severe hypertension). This status enables the detailed tracking of healthcare needs and plays a crucial role in health expenditure mapping and public health planning in France [33].

We found that there were small differences in the prevalence of chronic diseases not accounted for by matching. Interestingly, the most prominent difference was seen in the use of pain and inflammatory medication which could indicate a migraine co-pathology or secondary muscular pain syndromes in the case cohort. To verify the validity of our matching approach, we modelled the incremental costs attributable to vestibular disease, adjusting for other chronic diseases that could explain any differences. We found that even in individuals without major chronic diseases the cost difference was smaller but persisted.

By using the year 2022 for case definition and examining costs in 2023, our cost estimate is conservative, as it excludes the costs for the diagnosis or treatment of acute vestibular disease and instead reflects the ongoing burden of treatment and chronic care. It has been hypothesized that inadequately treated vestibular disease may become chronic and lead to long-term health problems, which our analyses may capture. We did not include 2022 costs in the main analysis because cases were selected based on vestibular medication and procedures, and thus acute costs would be expected to be higher in 2022. However, there is no clear reason why costs should remain elevated in 2023. Analysis of 2022 costs revealed significantly higher expenses for cases (€675.7 million), while the cumulative expenses for betahistine and acetylleucine in 2022 were only €1.9 million, and expenses for vestibular procedures were €8.7 million (see also Table S2). This results in over €656 million in additional expenses in 2022, which is similar to the incremental costs observed in 2023. Thus, incremental costs remain high over time, which may indicate either the chronic nature of the complaints or an inefficient diagnostic approach. Notably, the costs of specialist consultations and testing/imaging remain particularly high.

Cost data always reflect the health system being studied. The costs of vestibular disease found in our study are considerably lower than those reported in a large US study [20]. However, the US study used data from privately insured patients and extrapolated these costs to the total population, which may lead to an overestimation of the situation. The major advantage of our study is that it reflects the real cost burden on the French system, which can serve as an example for other European countries.

## Limitations

This study has several limitations inherent to the use of administrative health data.

First, the identification of vestibular disease relied on proxy indicators such as prescriptions and procedure codes, due to the absence of outpatient diagnostic coding in the SNDS. This may have led to misclassification and to both under- and overestimation of case status. However, the algorithm was designed to capture treated vestibular disease, not all prevalent vestibular symptoms or vestibular dysfunction. Its specificity is supported by the fact that betahistine and acetylleucine are primarily used for vertiginous disorders, and previous French national health insurance data have used betahistine prescriptions as an indicator of vestibular disease prevalence. Conversely, sensitivity is likely incomplete, as many patients with vestibular disorders, including patients with BPPV or conservatively managed disease, may not receive vestibular medication or coded procedures. For example, Seidel et al. reported that among 107,458 patients with dizziness- and vertigo-related diagnoses in German ENT practices, only 37% received at least one of the most frequently prescribed medications, namely antivertigo preparations or systemic corticosteroids [42]. Thus, our case definition is likely conservative and may underestimate the true population burden of vestibular disease. Procedure codes are highly compatible with vertiginous disease but are used much less frequently than medication prescriptions, as shown in Supplemental Table S2; therefore, separate medication-only and procedure-only analyses were not expected to meaningfully alter the main findings.

Second, although the matching procedure accounted for key confounders, residual confounding remains possible, particularly for factors such as functional status or undiagnosed conditions, which are not captured in the database. Although matching and regression adjustment accounted for major measured comorbidities, residual confounding cannot be excluded. The SNDS does not contain direct information on vestibular disease severity, symptom duration, functional status, frailty, health-related quality of life, or individual healthcare-seeking behavior. These factors may influence both the likelihood of receiving vestibular medication or procedures and subsequent healthcare expenditures. Consequently, part of the observed cost difference may reflect unmeasured differences in general health status, frailty, or care-seeking patterns rather than vestibular disease alone. Yet, the additional regression model showed that, even with the major other chronic diseases accounted for, patients with vestibular disease still had considerably more costs than those without.

Third, by excluding patients with certain chronic neurological diseases and those who died in 2022 or 2023, we may have introduced selection bias, potentially excluding severe or atypical cases of vestibular dysfunction. This confirms that our approach of prevalence estimation is very careful and conservative.

Fourth, cost attribution is based on a pharmaceutical cartography methodology, which, while standardized, may not fully capture costs associated with multimorbidity or shared resource use. To give an example, there were 11% of participants with diabetes, and we cannot fully rule out balance problems due to polyneuropathy. Still, this applies to both cases and controls, thus might not affect the difference.

Lastly, as we based our findings on two single years we cannot generalize findings beyond the specific study period.

To improve the accuracy of case identification, future studies could validate the proxy algorithm for vestibular disease using clinical cohorts or by linking with datasets that contain structured outpatient diagnoses, where available. Integrating patient-reported outcomes or specialist registries could also provide more detailed insights into symptom burden and disease severity. To address residual confounding, enhanced adjustment models that incorporate social, behavioral, or functional data - potentially derived from supplemental sources such as survey data or clinical records - may help reduce bias. Additionally, sensitivity analyses that include patients with chronic neurological diseases or those who died during follow-up could help assess the robustness of the findings regarding selection bias from exclusions.

Our analysis was restricted to direct reimbursed healthcare expenditures from the perspective of the French national health insurance system. We did not estimate indirect costs, including productivity losses or sick leave. Although the SNDS contains information on reimbursed daily sickness allowances for eligible beneficiaries, analysing work absence would require a dedicated study population restricted to persons of working age and with entitlement to sickness benefits, as well as careful distinction between sickness absence, occupational accidents or disease, maternity leave, and disability-related benefits. Such an analysis would therefore address a separate research question and was beyond the scope of the present study.

Vestibular disorders affect approximately 1 in 40 adults in France and are associated with significant excess healthcare costs. Patients with acute or chronic vestibular dysfunction often undergo extensive diagnostic evaluations and treatments that may not always be standardized or evidence based. Implementing consistent diagnostic pathways and optimizing therapeutic strategies, including medication use and referral practices, could reduce unnecessary healthcare expenditures and improve quality of care for patients experiencing dizziness, vertigo, and imbalance.

## Supplementary Information

Below is the link to the electronic supplementary material.

Supplementary Material 1

## Data Availability

Data is available upon request from the French Health Data Hub.
