# Supplementary material for: Prevalence and direct costs of vestibular disease in France – matched case-control study results from the 2022 and 2023 french health data system
Source: Cost Eff Resour Alloc. 2026 May 20;24:64. doi: 10.1186/s12962-026-00773-6 (PMC13191933; doi:10.1186/s12962-026-00773-6)
Supplement: Supplementary file 1 — Supplementary Material 1 [file 12962_2026_773_MOESM1_ESM.docx]

Supplementary information

Table S1. Population distribution of Metropolitan France (mainland and Corsica) ^41^

| Region |  |
| --- | --- |
| AUVERGNE RHONE ALPES | 12% |
| BOURGOGNE FRANCHE COMTE | 4% |
| BRETAGNE | 5% |
| CENTRE VAL DE LOIRE | 4% |
| CORSE | 1% |
| GRAND EST | 8% |
| HAUTS DE FRANCE | 9% |
| ILE DE FRANCE | 19% |
| NORMANDIE | 5% |
| NOUVELLE AQUITAINE | 9% |
| OCCITANIE | 9% |
| PAYS DE LA LOIRE | 6% |
| PROVENCE ALPES COTE D AZUR | 8% |

Table S2. Diagnostic or therapeutic procedures typical for the diagnosis or therapy of vestibular disease and used for case definition (https://www.assurance-maladie.ameli.fr/etudes-et-donnees/actes-techniques-ccam)

| Code | Description of procedure | Frequency of procedure in 2022 No. | Expenses in 2022 € |
| --- | --- | --- | --- |
| ADPA005 | Vestibular neurotomy, via translabyrinthine approach | 2 | 620 |
| ADPA005 | Vestibular neurotomy, via translabyrinthine approach | 2 | 1,479 |
| ADPA010 | Vestibular neurotomy, via retrolabyrinthine approach | 4 | 1,246 |
| ADPA010 | Vestibular neurotomy, via retrolabyrinthine approach | 4 | 3,054 |
| CERP001 | Calibrated bicaloric vestibular test with electronystagmography or videonystagmography | 9,356 | 351,131 |
| CERP002 | Vestibular rehabilitation session | 116,769 | 2,537,440 |
| CERP003 | Calibrated bicaloric vestibular test, pendular or rotatory, with electronystagmography or videonystagmography, and oculographic tests | 74,075 | 4,595,133 |
| CERP004 | Pendular or rotatory vestibular test with electronystagmography or videonystagmography | 21,172 | 846,260 |
| CERP005 | Calibrated bicaloric vestibular test without recording [clinical] | 7 | 0 |
| CEQP002 | Statokinetic measurement with stabilometry | 20 | 0 |
| CEQP005 | Analysis of static and/or dynamic vertical posture on a force platform [posturography] | 24 | 0 |
| CEQP008 | Recording of vestibular evoked potentials of saccular origin [otolithic evoked potentials] [saccule-collar evoked potentials] by electromyography | 7,115 | 207,644 |
| CCCA001 | Laser opening and occlusion of the posterior semicircular canal | 1 | 93 |
| CCCA001 | Laser opening and occlusion of the posterior semicircular canal | 1 | 237 |
| CCCA002 | Opening without laser and occlusion of the posterior semicircular canal | 0 |  |
| CCCA002 | Opening without laser and occlusion of the posterior semicircular canal | 1 | 474 |
| CCFA001 | Partial or total labyrinthectomy, via direct approach | 20 | 3,951 |
| CCFA001 | Partial or total labyrinthectomy, via direct approach | 29 | 12,270 |
| CCNB001 | Destruction of the labyrinth by injection of a pharmacological agent, via the transtympanic route | 39 | 2,174 |
| CCNB001 | Destruction of the labyrinth by injection of a pharmacological agent, via the transtympanic route | 282 | 6,403 |
| CCPA001 | Intralabyrinthine decompression via window approach, without laser | 1 | 221 |
| CCPA001 | Intralabyrinthine decompression via window approach, without laser | 2 | 661 |
| CCPA003 | Decompression or diversion of the endolymphatic sac | 18 | 3,167 |
| CCPA003 | Decompression or diversion of the endolymphatic sac | 23 | 9,173 |
| CCSA001 | Closure of perilymph fistula | 138 | 24,610 |
| CCSA001 | Closure of perilymph fistula | 176 | 54,139 |
|  | Total | 229,281 | 8,661,578 |

Table S3. Cost components for direct medical expenses in metropolitan France in 2022. Costs represent the total expenditures of the French national health insurance and do not include copayments or deductibles. All monetary values are reported in euros. Sums are shown without decimals; means and standard deviations are shown with two decimal places where applicable.

|  | Cases |  | Controls |  |
| --- | --- | --- | --- | --- |
|  | Sum | Mean (SD) | Sum | Mean (SD) |
| **Outpatient care** |  |  |  |  |
| General practitioner | 198,877,835 | 155.38 (147.87) | 132,363,014 | 103.41 (117.47) |
| Specialist | 375,865,082 | 293.65 (619.49) | 266,853,575 | 208.48 (624.38) |
| Dentist | 107,304,218 | 83.83 (180.09) | 94,543,932 | 73.86 (165.78) |
| Midwives | 4,944,248 | 3.86 (37.38) | 7,136,250 | 5.58 (54.09) |
| Physical therapy | 151,528,788 | 118.39 (334.11) | 102,716,505 | 80.25 (298.08) |
| Nursing | 228,683,081 | 178.66 (1117.81) | 223,587,918 | 174.68 (1158.86) |
| Other health professionals | 13,342,666 | 10.42 (73.53) | 8,362,802 | 6.53 (58.90) |
| Tests and imaging | 135,367,124 | 105.76 (134.07) | 111,614,882 | 87.2 (132.32) |
| Medication | 823,659,268 | 643.50 (3461.53) | 762,727,780 | 595.90 (3505.89) |
| Other health products | 214,517,407 | 167.6 (605.18) | 189,291,407 | 147.89 (640.79) |
| Transports | 120,686,514 | 94.29 (741.72) | 106,739,209 | 83.39 (765.05) |
| Other outpatient expenses | 16,734,440 | 13.07 (85.40) | 14,109,702 | 11.02 (73.48) |
| **Total outpatient services** | 2,391,510,671 | 1868.42 (4343.68) | 2,020,046,975 | 1578.20 (4412.51) |
| **Inpatient care** |  |  |  |  |
| General hospital | 1,373,752,998 | 1073.27 (4597.98) | 1,275,989,396 | 996.89 (4881.31) |
| Psychiatric hospital | 1,634 | 0 (0.80) | 0 | 0 (0) |
| Rehabilitation hospital | 144,006,435 | 112.51 (1273.81) | 143,532,123 | 112.14 (1430.85) |
| Nursing care at home | 128,08,775 | 10.01 (1050.06) | 18,210,545 | 14.23 (744.99) |
| **Total hospital/inpatient services** | 1,530,569,842 | 1195.79 (5149.6) | 1,437,732,064 | 1123.26 (5438.17) |
| **Total expenses** | 4,671,985,520 | 3650.09 (8100.68) | 3,996,269,726 | 3122.17 (8312.80) |

Table S4. Results of log-linear mixture model for 2023. An estimate of individual costs can be obtained by exponentiation of the estimate. For example, a woman with vestibular disease aged under 35 in the least deprived quintile living in Val de Loire would have annual costs of exp(7.88+1.17-0.50-0.06-0.24)= €1396

|  | Estimate | StdErr | ZValue | ProbZ |
| --- | --- | --- | --- | --- |
| Intercept | 7.88297215 | 0.00382891 | 2058.81 | 0.0000 |
| Vestibular disease present | 0.16575956 | 0.00159167 | 104.14 | 0.0000 |
| Sex male | 0.01570031 | 0.00177336 | 8.85 | 0.0000 |
| Age |  |  |  |  |
| 15-34 | -0.50206599 | 0.00351413 | -142.87 | 0.0000 |
| 35-54 | -0.3343078 | 0.0025683 | -130.17 | 0.0000 |
| 55-64 | -0.1693708 | 0.00256335 | -66.07 | 0.0000 |
| 65-74 | -0.28447251 | 0.00234386 | -121.37 | 0.0000 |
| 75+ | Reference |  |  |  |
| Quintile of deprivation |  |  |  |  |
| 1 | -0.06157818 | 0.0027491 | -22.40 | 0.0000 |
| 2 | -0.0322176 | 0.00255143 | -12.63 | 0.0000 |
| 3 | -0.0229586 | 0.00251175 | -9.14 | 0.0000 |
| 4 | -0.00930719 | 0.00245892 | -3.79 | 0.0002 |
| 5 | Reference |  |  |  |
| Region |  |  |  |  |
| AUVERGNE RHONE ALPES | -0.12144599 | 0.00347111 | -34.99 | 0.0000 |
| BOURGOGNE FRANCHE COMTE | -0.17636369 | 0.00453254 | -38.91 | 0.0000 |
| BRETAGNE | -0.18810072 | 0.00480995 | -39.11 | 0.0000 |
| CENTRE VAL DE LOIRE | -0.24345861 | 0.00473795 | -51.38 | 0.0000 |
| CORSE | 0.17869054 | 0.01059397 | 16.87 | 0.0000 |
| GRAND EST | -0.12780313 | 0.00366479 | -34.87 | 0.0000 |
| HAUTS DE FRANCE | -0.10371002 | 0.00369547 | -28.06 | 0.0000 |
| ILE DE FRANCE | -0.13040818 | 0.00331309 | -39.36 | 0.0000 |
| NORMANDIE | -0.18760763 | 0.0042479 | -44.16 | 0.0000 |
| NOUVELLE AQUITAINE | -0.17915098 | 0.00356452 | -50.26 | 0.0000 |
| OCCITANIE | -0.10697553 | 0.00359576 | -29.75 | 0.0000 |
| PAYS DE LA LOIRE | -0.20466265 | 0.00446355 | -45.85 | 0.0000 |
| PROVENCE ALPES COTE D AZUR | Reference |  |  |  |
| Comorbidity |  |  |  |  |
| Diabetes | 0.49414327 | 0.00368991 | 133.92 | 0.0000 |
| Cancers | 1.03647039 | 0.00274033 | 378.23 | 0.0000 |
| Chronic respiratory disease | 0.42203622 | 0.00289458 | 145.80 | 0.0000 |
| Chronic terminal kidney disease | 2.17869754 | 0.02016919 | 108.02 | 0.0000 |
| Inflammatory disease, HIV | 0.98082 | 0.00429486 | 228.37 | 0.0000 |
| Disease of liver or pancreas | 0.51043957 | 0.00762478 | 66.94 | 0.0000 |
| other ALD | 0.65449439 | 0.0040315 | 162.35 | 0.0000 |
| Pregnancy and birth | 0.8624497 | 0.00736901 | 117.04 | 0.0000 |
| Obesity treated in hospital | 0.42677941 | 0.00367132 | 116.25 | 0.0000 |
| Pain and antiinflammatory medication without other pathologies | 0.3783937 | 0.00530286 | 71.36 | 0.0000 |
| Cardiovascular disease | 0.5622347 | 0.00307856 | 182.63 | 0.0000 |
